# A Complete Pipeline to Extract Temperature from Thermal Images of Pigs

**DOI:** 10.3390/s25030643

**Published:** 2025-01-22

**Authors:** Rodania Bekhit, Inonge Reimert

**Affiliations:** Adaptation Physiology Group, Department of Animal Sciences, Wageningen University & Research, P.O. Box 338, 6700 AH Wageningen, The Netherlands; inonge.reimert@wur.nl

**Keywords:** thermal imaging, sensors, deep learning, segmentation models, classification models, pigs

## Abstract

Using deep learning or artificial intelligence (AI) in research with animals is a new interdisciplinary area of research. In this study, we have explored the potential of thermal imaging and AI in pig research. Thermal cameras play a vital role in obtaining and collecting a large amount of data, and AI has the capabilities of processing and extracting valuable information from these data. The amount of data collected using thermal imaging is huge, and automation techniques are therefore crucial to find a meaningful interpretation of the changes in temperature. In this paper, we present a complete pipeline to extract temperature automatically from a selected Region of Interest (ROI). This system consists of three stages: the first one checks whether the ROI is completely visible to observe the thermal temperature, and then the second stage uses an encoder–decoder structure of a convolution neural network to segment the ROI, if the condition was met at stage one. In the last stage, the maximum temperature is extracted and saved in an external file. The segmentation model showed good performance, with a mean Pixel Class accuracy of 92.3%, and a mean Intersection over Union of 87.1%. The extracted temperature observed by the model entirely matched the manually observed temperature. The system showed reliable results to be used independently without human intervention to determine the temperature in the selected ROI in pigs.

## 1. Introduction

Thermal imaging or infrared thermography is a contactless and non-invasive technique to remotely observe the temperature distribution patterns on the surface of the observed objects [1,2]. Thermal imaging creates images using the infrared waves that are emitted by all objects in the observed space. It has been widely used in different practical applications, including quality control [3], natural sciences [4], military [5], medicine [6,7,8], veterinary, and animal sciences [9]. In animal studies, it has shown great potential as a non-intrusive tool in remotely monitoring animals [2], or more specifically in monitoring their health [10]. Thermal cameras have the ability to capture and collect a huge amount of data remotely without causing stress to animals, which may alter the behavior of animals.

The aim of this study was to build a system able to detect and segment the body parts of pigs from thermal imaging footages to monitor the changes in the skin temperature of these parts to be used in future animal emotion research. The proposed system is composed of two integrated models. The first model is responsible for deciding whether the Region of Interest (ROI) is completely visible in the frame, and the second one segments the ROI to extract the maximum temperature. To our knowledge, this is the first study that used semantic segmentation to extract an animal body part from thermal videos, where the animals were kept in relatively low-lighting conditions, similar to the lighting conditions on real farms. The base of the ears was chosen as the ROI in this study, as it is proposed as an important area in emotion research [1,11], and it can be observed well from the top view, which is how the thermal camera was set up.

The main contributions of this study are as follows:

1. This study offers a complete pipeline able to automatically detect and track a Region of Interest (ROI) in the thermal videos of pigs moving freely. The pipeline can continuously extract temperature over a long period without any human intervention and save the observed temperature in external files for further analysis. To the best of the authors’ knowledge, this is the first complete pipeline for the automatic extraction of thermal temperature in animals.
2. The proposed system can be transferred to be applied on any animal or extended to extract the temperature of other body parts. In addition, thermal imaging is a suitable approach to identify animals, especially in low-light conditions, which is likely the case on most farms, whereas AI has the capabilities of processing and extracting valuable information from recorded data.
3. The system can be used to observe the change in the temperature of farm animals over a long period of time, which is crucial for animal research.

This paper is structured as follows: First, previous research related to our study is discussed in Section 2. The architecture of the system is described in Section 3. The dataset, preprocessing, and augmentation are subsequently described in Section 4. Section 5 presents the implementation of the system, and Section 6, the results. Finally, the results are discussed, and some conclusions are presented in Section 7.

## 2. Related Work

Thermal imaging has been used in animal research to monitor their physical condition and behavior. For disease diagnosis, for example, Dunbar et al. [12] detected temperature changes in the feet of mule deer infected with foot and mouth disease, finding a considerable rise in temperature two days before the first symptoms appeared. Avni-Magen et al. [13] found, during monitoring elephants with thermal cameras over three months, that the infected parts of the elephants such as the ears had a significantly higher temperature in comparison to other parts. In animal control research, thermal imaging was used to detect pregnancy in the black rhinoceros [14], and to determine the ovulation time in Asian elephants and black rhinoceros [15]. In animal emotion research, Nakayama et al. [16] reached to a conclusion that nasal temperature can work as an accurate indicator of a shift from a neutral to a negative emotional state in nonhuman primates. These studies relied mostly on the manual extraction of thermal data, as investigators manually located the area of interest (AOI) and extracted the temperature frame by frame. The manual method is a tiring and time-consuming task, considering that some thermal cameras record 20 to 30 frames per second. Hence, extracting the temperature manually for a 10 min thermal video, for instance, would be quite infeasible. Some recent studies used semi-automatic methods; however, they still depend on manual labor and a relatively subjective judgment to define the AOI [17]. Lu et al. [11] developed a Support Vector Machines (SVMs) algorithm based on the geometric shape and contour features of pigs to identify the ROI, i.e., the ear base. The model was applied on thermal images and compared with the manual measuring method. The comparison showed that for the left and right ear base, respectively, 97% and 98% of the testing images had an error within 0.4 °C. Although these results are great, the algorithm worked only on images selected by the experts in which the ROI was visible. The algorithm was not able to extract temperature automatically without supervision.

The advances in artificial intelligence and computer vision opened the door to process substantial amounts of data and automatize time-consuming tasks. However, there is still a limited number of thermal imaging studies where the potential of using artificial intelligence has been explored [17,18], especially in animal sciences. Cho et al. [19] proposed a deep learning model to automatically recognize peoples’ psychological stress levels from their breathing patterns using a thermal camera. Kakileti et al. [20] explored different architectures of semantic segmentation and found that encode–decode based architectures (UNet, VNet) work better in segmenting breast cancer in thermal images. Mazur-Milecka and Ruminski [21] used UNet and VNet models for the semantic segmentation of the thermal images of laboratory rats from their backgrounds, while they were in close contact during social behavior tests.

Image segmentation models have been widely used in medical image diagnosis, starting with fully convolutional networks (FCNs) [22] as a pioneering approach in the field of image segmentation. Further, other models have been developed like PSPNet [23], DeepLab [24], and Mask-RCNN [25] to improve the performance of image segmentation. UNet is the most popular network architecture and has been widely applied in medical research [26]. In our study, we are proposing a system consisting of two integrated models to extract the temperature of ROIs (i.e., the ear base) from thermal footage. The first model evaluates whether the base of the ear of both sides, left and right, is completely visible in the frame. If the ROI is visible, the second model segments the base of the ears to subsequently extract the maximum temperature.

## 3. Model Architecture

**Figure 1:**
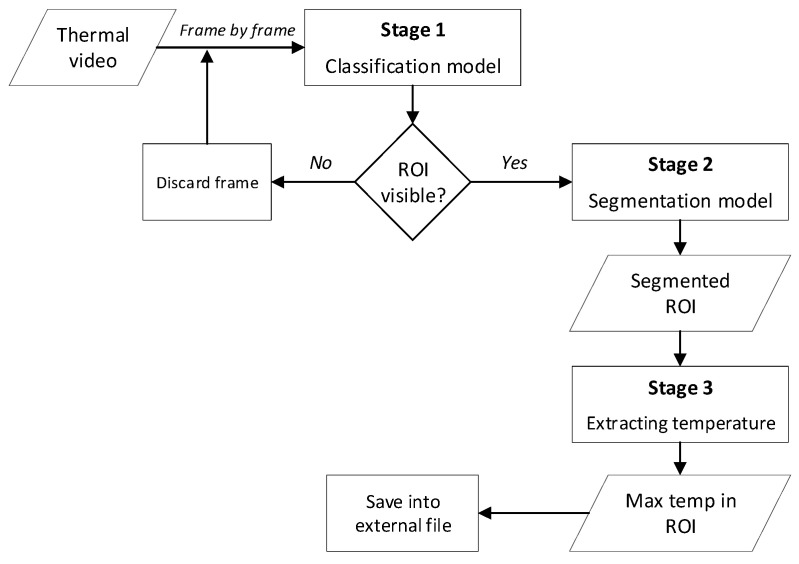
Flow chart of the complete model.

The proposed system for extracting temperature consists of three stages as shown in Figure 1. The first stage is a classification model, and each frame enters the model one by one as an input, where the model decides whether the ROI is completely visible in the frame. If the condition is met, the frame is passed to the next stage to extract the ROI. If the condition is not sufficient, the frame is discarded, and the same process is repeated for the next frame. The second stage is the segmentation stage, where the ROI is detected and extracted from the animal body. At the final stage, the maximum temperature is extracted and saved automatically with its number in an external file for further analysis.

### 3.1. Stage 1: Classification Model

**Figure 2:**
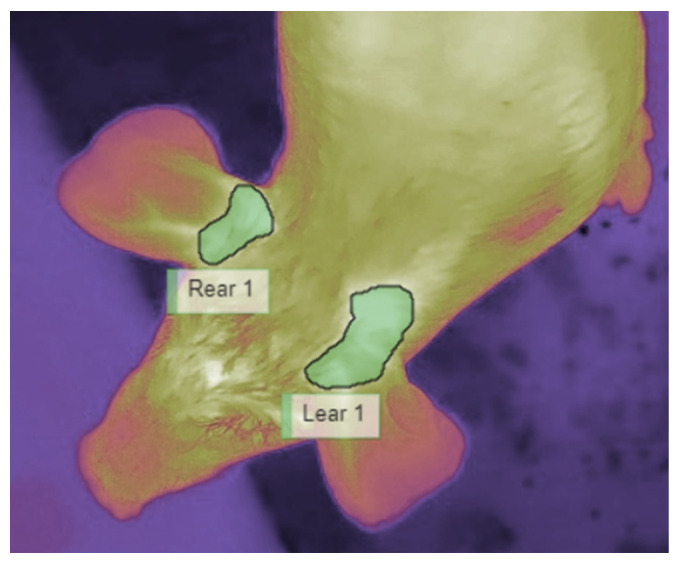
ROI is the ear base of both sides, left and right.

This stage is crucial to ensure the quality of the extracted temperature. The considered ROI in this study is the ear base of both left and right ears (Figure 2). As the animal moves freely in the pen during the test (see Section 4: dataset), these regions are sometimes partially visible or not visible at all in the frame. Skipping this stage and going directly to stage 2 may introduce errors in the extracted temperature, as the segmentation model will still be able to detect the partially visible ROI and extract the maximum temperature in the shown part only, whilst the real maximum temperature can be on the hidden part.

A common approach now in training a deep neural network is to use transfer learning rather than training the network from the beginning. Yosinski et al. [27] proved that even on applications that are far distant from the base task of the pre-trained network, transfer learning works better than using random initial values. The transfer learning approach was applied in training this classification model, using different architectures such as ResNet 50, VGG16, and Inception networks. As the main concern was to extract the temperature only when the ROI was completely visible in the frame, the architecture that yielded the least rate of false-positive results was chosen.

### 3.2. Stage 2: Segmentation Model

**Figure 3:**
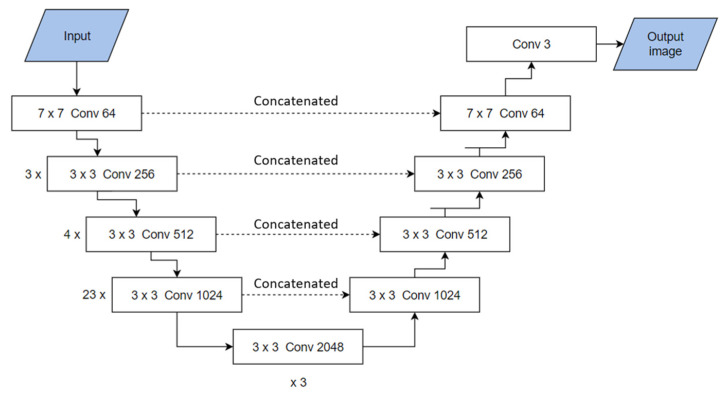
ResNet101-UNet architecture.

Semantic segmentation or image segmentation segments an input image by assigning each pixel to a certain label. It is a classification process operated at the pixel level [28]. The architecture of the UNet network is composed of two parts; encoder (down-sampling) and decoder (up-sampling). The encoder works on capturing the semantic information of the image, whereas the decoder locates this information. This architecture reduces the number of training parameters, enabling better performance. The model employed to segment the ROI is a UNet network with a backbone of a ResNet101 network as an encoder (Figure 3). He et al. [25] proposed the ResNet architecture in 2015, as a solution for the deep gradient degradation problem. Many subsequent networks followed with improving capabilities, such ResNet50 and ResNet101. It was inspired by the VGG-19 model [29], where a global average pool replaces the fully connected layer in the VGG model, and a “shortcut connection” is used. The backbone ResNet101 consists of a sequential of a 7 × 7 convolutional layer, a max pooling layer, and 33 residual blocks. Each residual block contains three 3 × 3 convolutional layers with a rectified linear unit (ReLU) activation and a batch normalization. The up-sampling part starts from a 1024-channel 24 × 32, processed by a 2 × 2 transposed convolution with a stride of 2. The up-sampled feature map, which has 512 channels and a size of 48 × 64, is concatenated with the corresponding feature map from the down-sampling path, which has been processed through a 1 × 1 convolution to produce a 512-channel output. This process is repeated until the size of the feature map is recovered with a size of 768 × 1024. The final layer is the output layer with a softmax activation function and three channels for the three detected labels; the ear base of the left side, the ear base of the right side, and anything else in the image frame is considered as background.

### 3.3. Stage 3: Temperature Extraction

At the final stage, the maximum temperatures [11,30] were extracted from the segmented ROIs. For each side, the thermal data of pixels outside the segmented area were discarded by assigning zero values to them, and then the maximum temperature was observed from the thermal pixels that only lay within the boundary of the extracted ROI. The maximum temperatures of both the left and right ear bases were then saved with the frame number in an external file.

## 4. Dataset

The thermal footages used for this research were captured during a frustration challenge test, which was part of a larger experiment to study resilience in pigs (see for more details [31]). The experiment was conducted at Carus, the animal research facility of Wageningen University and Research, Wageningen, the Netherlands. A total of 373 female pigs (TN70 × Tempo) were tested in the frustration challenge. For this challenge, each pig was taken out from its home pen and moved to a small pen (1.2 × 0.6 m) in a test room for 10 min. Here, the isolated animal was able to see, smell, and hear other pigs exploring and playing freely in a “play arena”. The inability to join the playing pigs may have induced a feeling of frustration. The experiment was approved by the Animal Care and Use Committee of Wageningen University and Research (DEC code: AVD1040020186245), and the established principles of laboratory animal care and use were followed, as well as the Dutch law on animal experiments.

A FLIR T1020 thermal imaging camera was mounted on a tripod with a distance of about 1 m between the pig’s head and the camera. The emissivity was set at 0.98. The ambient temperature and humidity of the test room were also set and checked regularly, and their settings were adapted when necessary. The resolution of the camera was 768, 1024 pixels, and had a 40 mm focal length lens with an accuracy of 0.02 reading at 25 °C and a thermal sensitivity range of −40 to 2000 °C. The thermal camera filmed the isolated pig in order to monitor if there was a change in temperature related to the induced negative emotional state.

A total of 373 thermal imaging videos were recorded during the test. To develop our model, 10 thermal videos were chosen to be processed with FLIR Research software Max 4.40 in order to build the datasets. The 10 videos were converted into both a jpg format and a csv format; the jpg images were used to train the model, and the csv format was used to extract thermal information.

**Figure 4:**
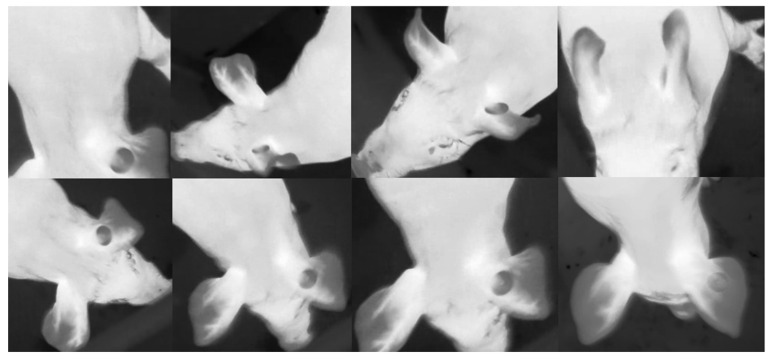
The upper row shows examples of unsuitable ROIs for extracting temperature, the bottom row shows examples where ROIs were appropriate to extract temperature.

For the classification model, a total of 12,784 images were selected from the processed 10 videos, assigning them to one of two classes; the ROI is visible (5388 images), and the ROI is not or partially visible (7396 images). Figure 4 shows examples of the two classes. The dataset is divided as follows; 9584 images for training, 1600 images for validation, and 1600 images for testing.

For the segmentation model, a new set of 577 images was selected to build the model dataset. The selected images were chosen to cover all possible positions of the ROI in order to be recognized by the model. The images were segmented manually using the APEER online platform. APEER is a cloud-based platform designed for image analysis tasks, offering various tools for annotating and segmenting images. The base of the pig’s ear lacked a well-defined boundary, as it varied between frames due to the pig’s movements. However, the area of interest consistently exhibited a higher temperature compared to other parts of the pig’s body, making it distinguishable in thermal imaging. Thermal images, like any digital images, consist of pixels, where each pixel represents a specific temperature value. The FLIR Research software assigns unique colors to these values based on the selected color palette. In this study, the IRONBOW color palette was used, which assigns lighter colors to higher temperature pixels. More information about thermal color representation palettes can be found at [32].

The 577 selected images were saved using this palette in RGB format. The lightest shades near the base of the ears, corresponding to the highest temperatures in this region, were identified as the boundary of the ROI. This selection was made by visually inspecting each image to locate these light regions. Switching off the red and green channels and keeping the blue channel helped in the visual assessment. To ensure consistency in defining the ROI boundaries, a single operator performed the annotation process using the brush tool provided in APEER. This protocol helped minimize variability caused by subjective judgment. The IRONBOW-colored images were used only to aid the annotation process. The training of the segmentation model and all predictions were performed on grayscale images. Each annotated image has three labels; left-ear-base side, right-ear-base side, and the background. Due to the high cost of the annotation process in terms of time and effort, there are always insufficient data to support segmentation models’ training. Data augmentation helps to expand the annotated dataset to improve model robustness and reduce the possibility of overfitting during the training process [33,34]. To this extent, the annotated images were flipped horizontally and vertically and saved in new files, making a total number of 1731 images. Other augmentation processes were performed during training, such as random rotation, shifting, and different contrast and brightness. The dataset was split into 1431 images for training, 200 images for validation, and 100 images for testing.

## 5. Model Implementation

For training and testing of the proposed model, an HP workstation with 2× Intel Xeon E5-2678V3, NVIDIA Quadro RTX 5000 graphic card, and 128 GB of RAM (sourced from a computer hardware seller in the Netherlands) was used along with a Tensorflow 2.10 and Python 3.9.0 environment. In this section, the implementation of each stage is explained.

### 5.1. Classification Model

The images were resized to 320 × 320 and used as an input to train the model. Different network architectures were trained, and the network with the lowest false-positive rate was chosen. The summary of the hyperparameters of the model is shown in Table 1. The model is trained with the Stochastic gradient descent (SGD) optimizer, momentum 0.9, and learning rate 1 × 10^−3^, batch size 32, epochs 300, and callbacks is ReduceLROnPlateau (factor = 0.8, patience = 6) monitoring the valid loss. To calculate the loss of the model, the binary cross-entropy loss function was used. The loss function is expressed as follows:

(1) $$Loss=\frac{1}{n}(\sum_{i=1}^{N}-y^{\left(i\right)}\log\left({\hat{y}}^{\left(i\right)}\right)-\left(1-y^{\left(i\right)}\right)\log\;(1-{\hat{y}}^{\left(i\right)}))$$

where *N* is the number of classes, y is the real label, and $\hat{y}$ is the predicted label for an image (*i*).

The metrics used to evaluate the performance of the model are the accuracy and false-positive rate (*FPR*). The accuracy indicates the overall performance of the model. It calculates the percentage of correct classification, using the following function:

(2) $$Accuracy=\frac{TP+TN}{TP+TN+FN+FP}$$

where TP, true positive, is the number of labels correctly predicted as positive; TN, true negative, is the number of labels correctly predicted as negative; FP, false positive, is the number of images falsely predicted as positive; and FN, false negative, is the number of images falsely predicted as negative. For this study, positive means that the ROI is well and completely visible, and negative means that the ROI is not or only partly visible.

The false-positive rate refers the ratio of images misclassified to be positive to the sum of rightly classified images. It is calculated as follows:

(3) $$FPR=\frac{FP}{TN+FP}$$

where TN and FN denote similarly as above.

### 5.2. Segmentation Model

The input image size is its original size (1024 × 768), as the segmented pixels were used to retrieve the thermal information from the original thermal file, and any resizing would cause information loss. The network was trained using the Stochastic gradient descent (SGD) optimizer with a batch size of 2. The initial learning rate was 1 × 10^−4^, with a learning rate reduction if no improvement occurred in the network performance for 10 epochs. A summary of the hyperparameters of the model is shown in Table 2. The Jaccard loss was used as a loss function in training. The Jaccard index, also known as the Jaccard similarity coefficient, was introduced in Jaccard [35]. It is one of the most frequently used loss measures in segmentation models [36]. It measures the similarity between two sets. In segmentation models, the loss function evaluates the dissimilarity between the ground truth and the predicted segmentation value. The Jaccard loss is calculated as follows:

(4) $$Jaccard\;loss=1-IoU=1-\frac{Area\;of\;Overlap}{Area\;of\;Union}$$

where IoU is the Intersection over Union. It measures the ratio of the intersection and union of the predicted pixels ${\hat{y}}_{i}$ and ground truth $y_{i}$.

The performance of the model was evaluated using Pixel Class Accuracy and mean Intersection over Union (*mIoU*). Pixel Class Accuracy (*PCA*) measures the average of the proportion of the total number of correct pixels predicted by the model for each class, which is given by the following:

(5) $$PCA=\frac{1}{N}\;\sum_{i=1}^{N}\frac{P_{c_{i}}}{P_{t_{i}}}=\frac{1}{N\;}\sum_{i=1}^{N}\;\frac{{TP}_{i}+{TN}_{i}}{{TP}_{i}+{TN}_{i}+{FP}_{i}+{FN}_{i}}$$

where $P_{c_{i}}$ is the number of correctly predicted pixels in class i, $P_{t_{i}}$ is the total number of pixels in that class, and N is the total number of classes. ${TP}_{i}$ is true positive and denotes to the number of pixels rightly predicted as a class i, ${TN}_{i}$ is true negative and denotes to the number of pixels rightly predicted as not a class i, ${FP}_{i}$ is false positive and represents the number of pixels falsely predicted as a class i, and ${FN}_{i}$ is false negative and represents the number of pixels falsely predicted as not a class i.

Although Pixel Class Accuracy can give an indication about model performance, it is not sufficient in segmentation models, due to a class imbalance problem, where there is a dominant class (background), and the classes needed to be predicted cover only a small portion of the image. Hence, the model could show high accuracy as it predicts all pixels as the dominant class, where in reality, it shows poor results to predict other classes. Therefore, mean Intersection over Union (*mIoU*) is crucial to assess the performance of segmentation models. The *IoU* is calculated as follows:

(6) $$mIoU=\frac{1}{N}\;\sum_{i=1}^{N}\;\frac{Area\;of\;{Overlap}_{\;i}}{Area\;of\;{Union}_{\;i}}=\frac{1}{N}\;\sum_{i=1}^{N}\;\;\frac{{TP}_{i}}{{TP}_{i}+{FP}_{i}+{FN}_{i}}$$

where Area of Overlap i is the number of overlapping pixels between the prediction and the ground truth (${TP}_{i}$) in class i, and Area of Union i is the sum of the predicted pixels and the ground truth pixels in the same class, including ${TP}_{i}$, ${FP}_{i}$, and ${FN}_{i}$ [37]. *N* is the number of classes.

### 5.3. Temperature Extraction

The final stage of the model was to process the entire thermal video and extract the maximum temperature. This stage worked like the engine of the model; it used the previous two models to extract and save the temperature. For each frame in the sequence of a thermal video, the thermal frame was converted to a jpg image format and resized to an image size of 320 × 320. The classification model examined the visibility of the ROI. If the condition was met, the segmentation model segmented the ROI of both sides and forwarded it to the final stage of the model to extract the temperature. The maximum temperatures of both sides with their coordinates were saved as records in a csv file along with the frame number. If the ROI was not completely visible in the frame, the frame was discarded, and its record was saved as a missing record. Then, the next frame went through the same process. At the end of the thermal video, the file was saved externally on a hard drive to be analyzed for further research. Each thermal footage had a duration of over 10 min. After processing by the model, more than 19,000 records were saved per video. Table 3 shows an example of the saved temperature.

The temperature was measured manually as well, and these manual records were compared to the observed measurements by the model (see Section 5 for more information).

## 6. Results

The performance of the different architectures of classification models was examined on the test dataset, and a comparison of their performance is shown in Table 4. Most architectures achieved a high overall accuracy; however, the Inception and ResNet50 networks achieved the best results in terms of the lowest proportion of falsely predicted positives (*FPR*s), which was our concern in this study. Model ensembling is a method that combines several individual models to achieve better generalization performance [38]. The Inception and ResNet50 were ensembled equally, which achieved a slightly better performance, with an overall accuracy of 99% and an FPR of 0.5%.

**Figure 5:**
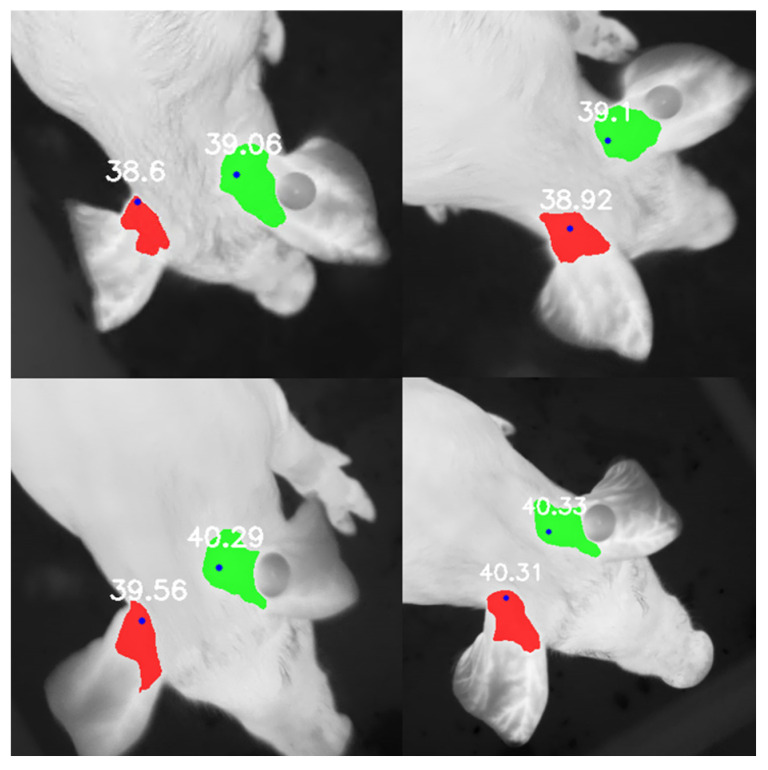
Examples of automatically extracted temperatures by the model.

For the segmentation model, the overall PCA was 92.3%, and for the left-side and right-side classes, the PCA was 88.7% and 88.2%, respectively. The overall mIoU was 87.1%. The IoU for the left side was 80.9%, and for the right side, it was 80.5%. Figure 5 shows four examples of the segmentation model output.

**Figure 6:**
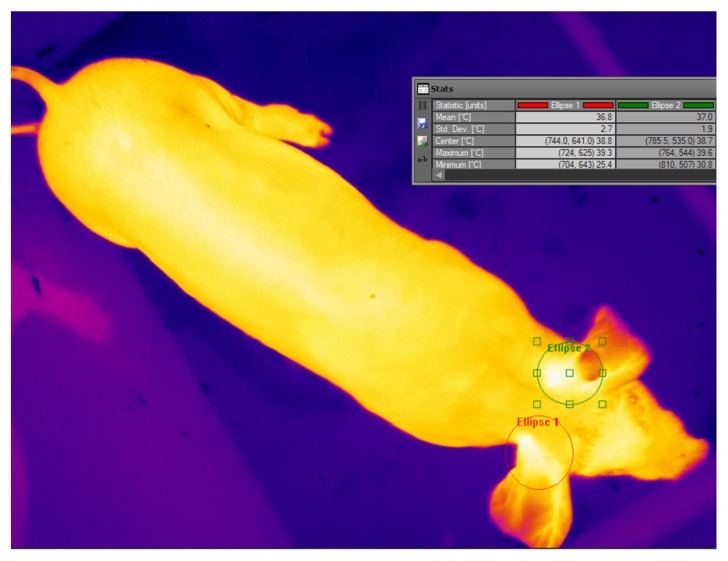
The temperatures of the ear base of the left and right side were measured manually using FLIR ResearchIR. Ellipse 1, red in the image, was for the right side, and Ellipse 2, green in the image, was for the left side. Both ellipses were drawn manually by the observer. The statistics of the ellipses are shown in the table within the figure.

In addition, the model was tested by comparing the model temperature output with manual temperature measurements. A set of 200 images was measured manually using FLIR ResearchIR, version 4.40. The temperature parameters of the left and right ear base areas can be obtained by drawing ellipses around each ear base position, as shown in Figure 6. FLIR subsequently provided temperature statistics of the drawn Region of Interest, including the mean, minimum, and maximum temperatures.

**Figure 7:**
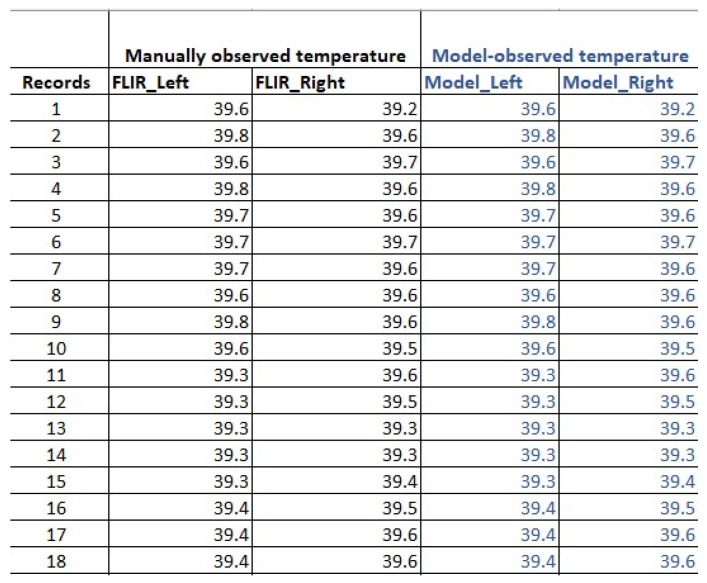
Comparison between manually observed temperature and temperatures observed by the model.

For all 200 records, the manually measured temperatures and the model output temperatures had 100% agreement together. Figure 7 presents a comparison of selected records, showing the manual temperature observations alongside the model’s output temperatures.

## 7. Discussion and Conclusions

Thermal imaging as a non-invasive technique to monitor the health and welfare of animals has become increasingly popular. However, it is a labor-intensive technique; therefore, automation is key to achieve a functioning thermal system. In this study, we described the development of an automatic system to process thermal videos and extract temperature automatically for further analysis. The model is designed to extract the external body temperature of a defined body part of pigs. The defined area was the ear base of both sides, left and right. The emotions of animals are regarded to consist of two dimensions, i.e., valence (an emotion is positive or negative) and arousal (intensity of the emotion) [39,40,41]. Skin temperature can likely be used as an indicator of valence and arousal in both humans and animals [42,43,44]. When aroused by a stimulus, the activation of the sympathetic branch of the autonomic nervous system leads to peripheral blood vessels to constrict in order to direct blood and, thereby, energy and oxygen to the core of the body where it is needed [45,46]. This leads to an initial drop, and subsequently, due to vasodilation, to a gradual increase in temperature in the periphery of the body, such as (parts of) the face [45,46]. Neuroimaging research, moreover, suggests that the two hemispheres of the brain might play different roles in processing positive vs. negative emotions, with a debate on their specific contribution [47,48,49,50]. The asymmetry in, for instance, ear skin temperature in response to an emotional stimulus may, thus, reflect lateralized brain activity and hence be a marker of valence [44]. For this context, the developed system could be used in future research to process thermal video footages of pigs in the frustration challenge test to extract the maximum temperature of the base of the ears every 10 s to examine the hypothesis of asymmetry in ear temperature as a response to a negative stimulus. The proposed system consists of three stages. The first one uses a classification model to check the visibility of the ROI. The accuracy of this classification model was 99% and had a false-positive rate of 0.5%. The second stage tracks and segments the ROI, using a UNet network with a backbone of a ResNet101 network as an encoder. The overall PCA of this segmentation model was 92.3%. For the left-side and right-side classes, the PCA was 88.7% and 88.2%, respectively. The overall mIoU was 87.1%. The IoU for the left side was 80.9%, and for the right side, it was 80.5%. We consider the performance of the model as good, especially for an ROI that did not have clearly defined boundaries. Future work could explore improving performance by evaluating alternative segmentation architectures, such as using UNet with different backbone networks, or exploring models like fully convolutional networks (FCNs) [22], SegNet [51], and DeepLabV3+ [52], as well as testing different protocols for defining the area of interest. Additionally, experimenting with alternative annotation strategies or incorporating more training data could further enhance model performance. The final stage extracts the maximum temperature and saves it in an external file. For this study, the ROIs were selected to be the left and right side of the ear base of pigs.

The performance of the model was also examined by comparing the temperature in a set of 200 thermal images measured manually and by the model. The comparison showed full agreement between the manually observed records and the model-observed records, showing that the model can be used reliably and as a replacement for the manual method to obtain the temperature from future thermal records.

The model was well able to track and locate the ROIs in videos recorded in relatively low-lighting conditions, which are similar to the conditions on farms. Thermal cameras indeed have the ability to record high-quality thermal footages in ill-light conditions [53,54], which thus shows their potential to be used in high-precision farms.

Unlike in human research, the incapability of animals to pose to the camera added a new challenge for any automated process to be applied on animals. The model developed in this study tackled this problem by examining first the visibility of the ROIs and observing the temperature only when the ROI was completely visible in the thermal frame. This also means that this automatic process can work to monitor the temperature of the selected ROI of an animal over a long period of time without any human intervention.

Although the pig was able to move freely in this study, it was confined alone in a small pen as a negative stimulus. The model’s performance, therefore, still needs to be examined in conditions where pigs have more freedom to move and have close interactions with each other, which is the normal situation on a farm. Some modifications may be needed to accommodate the new filming situation, which can be investigated in future research.

The chosen area of interest in this study does not have a well-defined boundary, such as those found in more distinct features like the eyes or nose. It is subjectively defined, even for manual observation. To ensure consistency in defining the boundaries of the ROI, the annotation process was carried out by a single operator, following a detailed protocol described in the methodology. However, using multiple operators may introduce variability in the boundary definition, potentially impacting the accuracy of the segmentation model. Future research could evaluate the extent of this variability by involving multiple operators and examining its effect on the model’s performance.

The methodology presented here can, moreover, be applied in different fields in animal husbandry, such as health, by monitoring the temperature of the eyes, feet, and ears of cattle and pigs [55,56]. Furthermore, observing changes in the body surface temperatures of specific areas, such as the armpits, buttocks, chest, and groin, in animals requiring high physical performance (e.g., horses) can provide valuable insights into body temperature regulation during training. This information can be useful for assessing horse fitness [57,58]. A last example is that changes in vulva temperature can be used to identify estrus and monitor the transition toward ovulation [59,60,61]. Other examples can be found in [62,63].

In conclusion, the model developed in this study showed to be reliable to determine the temperature in the selected ROI in pigs without human intervention. With some more research, this model can likely even be used, for instance, to monitor temperature changes in interacting pigs and in other research areas in animal science that make use of thermal imaging.

## Figures and Tables

**Table 1:** Summary of the hyperparameters of the classification model.

	Parameter Name	Selected Value
Model setting	Input size	320 × 320 × 3
	Optimizer	SGD
	Learning rate	1 × 10^−3^
	Momentum	0.9
Training setting	Loss function	Binary cross-entropy
	Batch size	32
	Epoch	300
ReduceLROnPlateau	Monitor	Valid loss
	Patience	6
	Factor	0.8
Environment	GPU	NVIDIA Quadro RTX 5000
	Platform	Python 3.9
	Tool box	Tensorflow

**Table 2:** Summary of the hyperparameters of the segmentation model.

	Parameter Name	Selected Value
Model setting	Input size	768 × 1024 × 3
	Optimizer	SGD
	Learning rate	1 × 10^−4^
	Momentum	0.9
Training setting	Loss function	Jaccard loss
	Batch size	2
	Epoch	300
ReduceLROnPlateau	Monitor	Valid loss
	Patience	10
	Factor	0.8
Environment	GPU	NVIDIA Quadro RTX 5000
	Platform	Python 3.9
	Tool box	Tensorflow

**Table 3:** An example of extracted temperature.

Frame	Left Temp ^1^	Right Temp	L_pos ^2^	R_pos
0	39.83	39.48	(652, 667)	(724, 614)
1	39.78	39.45	(631, 673)	(718, 619)
2	39.56	39.38	(623, 679)	(709, 636)
3	39.53	39.24	(615, 690)	(694, 649)
4	39.55	39.20	(599, 698)	(682, 660)
5	39.39	39.20	(576, 712)	(665, 674)

^1^ The left temp column gives the extracted maximum temperature of the base of the left ear, and similarly, the right temp column is for the right side of the ear. ^2^ The L_pos column shows the coordinates of the pixel with the maximum temperature in the left side, and similarly R_pos are the coordinates for the right side.

**Table 4:** A comparison between different model architectures’ performance.

Model Architecture	Accuracy	False-Positive Rate
ResNet-50	97.4%	1.62%
VGG-16	96.9%	2.12%
Inception	97.7%	1.52%
ResNet-101	95.8%	2.88%
Xception	96.8%	2.67%

## Data Availability

Dataset available on request from the authors.
